# Solution-Based Processing for Scaffold Fabrication in Tissue Engineering Applications: A Brief Review

**DOI:** 10.3390/polym13132041

**Published:** 2021-06-22

**Authors:** Elisa Capuana, Francesco Lopresti, Francesco Carfì Pavia, Valerio Brucato, Vincenzo La Carrubba

**Affiliations:** 1 Department of Engineering, University of Palermo, RU INSTM, Viale delle Scienze, 90128 Palermo, Italy; elisa.capuana@unipa.it (E.C.); francesco.lopresti01@unipa.it (F.L.); francesco.carfipavia@unipa.it (F.C.P.); valerio.brucato@unipa.it (V.B.); 2 ATeN Center, University of Palermo, Viale delle Scienze, 90128 Palermo, Italy

**Keywords:** scaffold, tissue engineering, processing, electrospinning, phase separation, freeze-drying

## Abstract

The fabrication of 3D scaffolds is under wide investigation in tissue engineering (TE) because of its incessant development of new advanced technologies and the improvement of traditional processes. Currently, scientific and clinical research focuses on scaffold characterization to restore the function of missing or damaged tissues. A key for suitable scaffold production is the guarantee of an interconnected porous structure that allows the cells to grow as in native tissue. The fabrication techniques should meet the appropriate requirements, including feasible reproducibility and time- and cost-effective assets. This is necessary for easy processability, which is associated with the large range of biomaterials supporting the use of fabrication technologies. This paper presents a review of scaffold fabrication methods starting from polymer solutions that provide highly porous structures under controlled process parameters. In this review, general information of solution-based technologies, including freeze-drying, thermally or diffusion induced phase separation (TIPS or DIPS), and electrospinning, are presented, along with an overview of their technological strategies and applications. Furthermore, the differences in the fabricated constructs in terms of pore size and distribution, porosity, morphology, and mechanical and biological properties, are clarified and critically reviewed. Then, the combination of these techniques for obtaining scaffolds is described, offering the advantages of mimicking the unique architecture of tissues and organs that are intrinsically difficult to design.

## 1. Introduction

Tissue engineering (TE) is an emerging biomedical engineering discipline that combines cells, materials, and biochemical factors to restore or replace different types of biological tissues [1].

In this context, the fabrication of biopolymeric scaffolds with an appropriate porous structure for cell growth guidance in three dimensions while mimicking the complex architecture of native tissues is a key challenge. To process synthetic and natural biomaterials into porous scaffolds, several techniques starting from polymer solutions or polymer melts were proposed. Melt-based technologies offer the possibility to avoid the use of organic solvents that can elicit cytotoxic effects on the final structure. However, melt-based methods normally produce constructs exhibiting a porosity lower than the range recommended in the literature for TE applications (i.e., lower than 80%) [2].

On the other hand, 3D scaffolds produced by solution-based technologies provide highly porous structures for tissue restoration and cell growth, hence facilitating the TE challenge in the development of functional and biocompatible scaffolds. Furthermore, these approaches permit obtaining a wide plethora of porous structures depending upon the particular process adopted. The main drawback of solution-based methodologies relies on the dissolution of the polymeric chains in organic solvents potentially harmful for cells [3]. For this reason, solvent removal efficiency is a key parameter for the final product quality [4,5,6].

**Figure 1:**
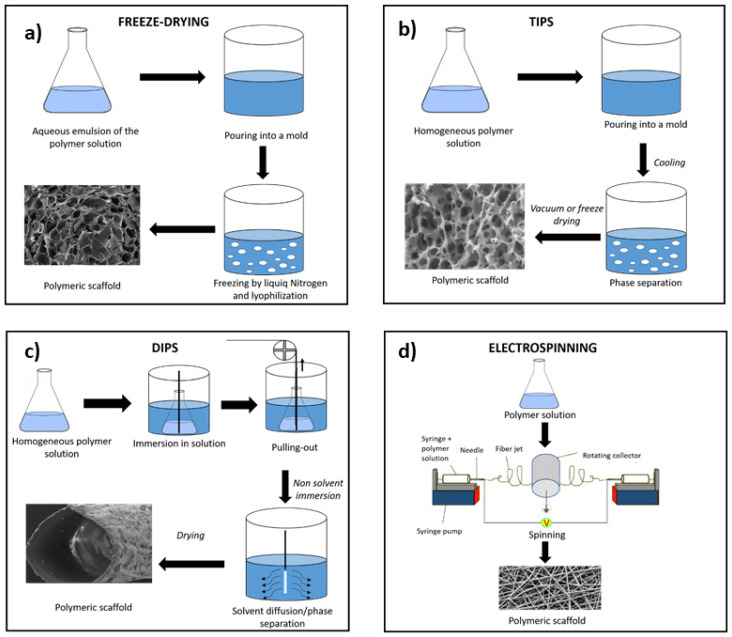
Schematic illustration of the solution-based fabrication process of scaffolds: (**a**) freeze-drying, (**b**) thermally induced phase separation, (**c**) diffusion induced phase separation, and (**d**) electrospinning.

Nowadays, several fields of research are giving remarkable attention to the fabrication via solution-based technologies, providing scaffolds in which cells can attach and proliferate to support tissue repair and hence the regeneration of the native biological structures. With this respect, the accessibility of a relatively wide range of biomaterials further supports the use of these technologies. Biocompatibility, biodegradability, appropriate mechanical properties, and high surface-to-volume ratio are the main characteristics of materials suitable for TE treatments [7,8]. Generally, the choice of the material for solution-based technologies relies on the desired morphology, function, and application of the produced scaffold, including biopolymers such as polycaprolactone (PCL) [9,10], polylactide (PLA) [11,12,13], poly(lactide-co-glycolide; PLGA) [14], poly(vinyl) alcohol (PVA) [15], gelatin [16], chitosan [10], and collagen [17,18]. According to the expected structural and mechanical performance for tissue replacement, different solution-based fabrication methods could be applied (Figure 1), such as freeze-drying [17,19,20] (Figure 1a), Thermally or Diffusion Induced Phase Separation (TIPS (Figure 1b) or DIPS (Figure 1c)) [21,22], electrospinning [23,24] (Figure 1d), or a valuable combination of them [25,26,27,28].

Recently, many studies have focused on the effect of the pore structure on cell organization and tissue regeneration [29,30]. As compared to low porosity and small average pore size matrices, high porosity and larger pore size enhance the formation of 3D large cells aggregate among the fibers/pores [31]. In scaffolds with low porosity and small pore dimension, cells tended to spread as individual cells on a single fiber surface or bridged across several fibers/pores [32]. Two types of structures can be obtained by solution-based technologies: foam-like scaffold or fibrous scaffold. Foams can be fabricated by freeze-drying and phase separation by finely tuning porosity, pore size, and shape while having a completely interconnected pore network. Fiber scaffolds can be produced by electrospinning, creating fibers that are deposited on top of each other in a random or aligned manner [33].

3D functional scaffolds can be produced using both natural-based or synthetic-based materials [34]. Synthetic-based scaffolds can be reproducible and readily available and are cheap to fabricate, offering control over many critical properties, such as the degradation rate and mechanical properties. Natural biomaterials better support cell attachment, growth, and differentiation, since they induce a more positive response of the host tissue and do not produce harmful degradation products, enhancing the integration within the body [35]. Apart from these materials, solution-based manufacturing allows the exploitation of molecules to enhance cell activity [36,37]. Traditionally, ceramics, active agents, and growth factors were integrated within the formulated scaffolds for more rapid tissue growth or increased compatibility [4]. Specifically, the additive is added to the solution/dispersion, and a bioactive matrix is produced with enhanced structural and biological performances. Hence, solution-based technologies have the potential to test several combinations of scaffold, cells, and bioactive molecules to facilitate the regeneration of damaged tissues.

This review article is an inclusive overview of the different solution-based fabrication techniques for tissue engineering, the numerous biomaterials that can be used, along with the advantages and limitations in scaffold manufacturing. Although this topic can be extensively reviewed in the literature, this paper is specifically a focused study of solution-based technologies, their newest advancements in terms of methods and materials, and the related emerging hybrid manufacturing processes.

Moreover, the final properties of the produced scaffolds are illustrated to discuss the design parameters affecting the ability of the constructs to be used for in vitro testing and tissue regeneration.

## 2. Freeze-Drying

**Figure 2:**
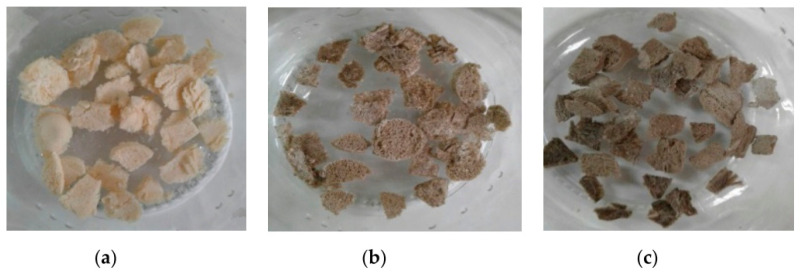
Freeze-dried scaffolds of chitosan (CS) (**a**) without graphene oxide (GO), (**b**) with 0.5% of GO, and (**c**) with 1.0% of GO [43]. Reprint with permission from MDPI.

Freeze-drying (or lyophilization) is a conventional technique for 3D scaffold fabrication, creating a complex scaffold geometry while achieving a uniform pore morphology [38]. This method converts solutions into solids through four steps: pretreatment, freezing, primary drying, and secondary drying [39]. In the first phase, treatments are applied to improve the precursors’ stability during the process, driving the prepared precursors to be ready for the freezing step. In this latter, the solution is cooled down to a temperature below the triple point of the solvent so that solvent crystals sublimate and the polymer creates an arranged network within the interstitial spaces [40]. The final drying phase is divided into two steps in which the solvent in frozen and unfrozen components are removed by sublimation and evaporation, respectively [41]. Temperature and pressure cycles are accurately adjusted during the whole process to avoid structural damages of produced scaffolds and achieve a controlled interconnected porous architecture. The feasibility of freeze-drying to fabricate porous uniform structures of polysaccharides-based hydrogel was investigated by Grenier et al. [42]. While examining the mechanism of pore formation during freezing, it was found that secondary nucleation was responsible for the formation of most ice grains and polymer macro-network into the inter-granular space. The results of a homogenous cell seeding from the culture of pre-osteoblastic cells validated the adequately interconnected pores of the produced freeze-dried scaffolds. Valencia et al. [43] also employed freeze-drying in the fabrication of chitosan scaffolds enriched with graphene oxide (GO) and demonstrated their biocompatibility and tissue recovery after 30 days of scaffold implantation in rats’ skin. At each tested GO percentage, homogeneous structures with appropriate porosity and roughness for cell growth were fabricated, as shown in Figure 2.

Recently, Mesgar et al. [44] produced gelatin (G)/chitosan (Ch) scaffolds with a reinforcement of functionalized multiwall carbon nanotubes (f-MWCNTs) by freeze-drying. They used a compressive strength test and a scanning electron microscope (SEM) to compare porosity, compressive modulus, pore size, and f-MWCNTs dispersion of each fraction of nanotubes added to the scaffold sample, delighting the attainment of the strongest reinforced scaffolds at moderate reinforcement levels. In another approach, Chen et al. [17] evaluated the osteoconductivity into collagen (Col)/hydroxyapatite(HA) hybrid scaffolds fabricated by freeze-drying at different HA concentrations, all of which enhanced bone marrow mesenchymal stem cells (BMSCs) osteogenic differentiation compared to the pure collagen scaffold. Recently, a novel “twice freeze-drying” method was used by Zhai et al. to fabricate a three-layer scaffold to mimic the cartilage layer, isolation layer, and subchondral bone layer for the repair of osteochondral defects [45]. By using chitosan, gelatin, and beta-tricalcium phosphate (β-TCP), the authors obtained scaffold layers with controlled pore size, suggesting that the properties of the scaffolds are not affected by freeze-drying in two subsequent steps.

**Figure 3:**
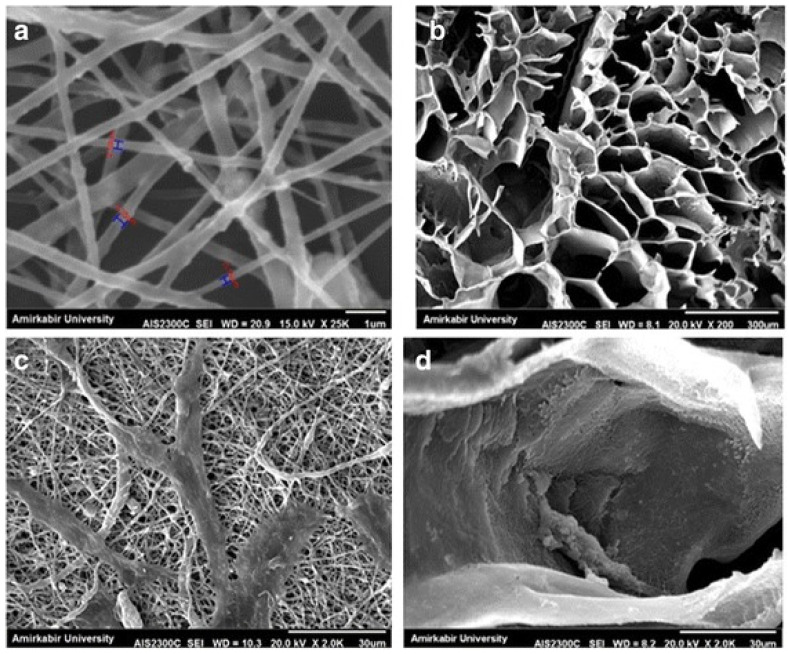
SEM images: (**a**) 1 μm; electrospinning, (**b**) 300 μm; freeze-drying, obtained from the surface of the synthesized scaffolds, (**c**) cell attachment, 30 μm; electrospinning, (**d**) 30 μm; freeze-drying [49]. Reprint with permission from PMC.

Similar to natural scaffolds, synthetic scaffolds have been fabricated via freeze-drying from polymer solutions to produce highly porous 3D scaffolds with complex pore morphology [46]. Recently, Dattola et al. [47] developed a poly(vinyl) alcohol (PVA) scaffold for cardiac tissue engineering by using a combination of freeze-drying and gas foaming processes. Specifically, the freeze-drying method was used to cross-link PVA foam without the use of any cross-linking agents, hence, improving the scaffold biocompatibility. PVA was also utilized in the freeze-drying technique to produce a bilayer scaffold containing cellulose nanofiber with a highly interconnected porosity for engineering skin regeneration [20]. By varying the polymer concentration, scaffolds were obtained with a range of porosities and pore sizes close to epidermis and dermis. The scaffolds seeded with fibroblasts supported a good biological response in terms of cell viability. On the other hand, the low hydrophilicity of synthetic polymers reduces their cell affinity, which, however, can be enhanced by incorporating natural biopolymers that promote cell adhesion due to their natural biocompatibility. This feature can be successfully obtained by freeze-drying technique, as demonstrated by the outcomes of Mozafari et al.’s work, studying the effect of different collagen concentrations on the biological and mechanical properties of poly(ε-caprolactone) (PCL)-collagen scaffolds [48]. The addition of natural ceramic materials to scaffold architecture is another strategy for improving their biocompatibility and bioactivity. The advantage of freeze-drying in producing such a scaffold with appropriate physical and biological performances was validated by Namini et al., who compared the properties of freeze-dried and electrospun polylactide-co-glycolide/hydroxyapatite (PLGA/HA) scaffolds [49]. The freeze-dried PLGA/HA scaffold showed higher cell viability and cell attachment than those on the electrospun PLGA/HA scaffold on day 7 of being seeded, as shown in Figure 3.

According to the authors, this result is related to the porous structure of freeze-dried scaffolds showing larger pore size, higher porosity, and interconnection rather than the fibrous structure of the electrospun PLGA/HA constructs.

Overall, freeze-drying can produce pore structures that can be effectively controlled by modifying the freeze-drying cycle, hence fabricating a variety of scaffolds with a wide range of mean pore dimensions [50]. Moreover, by circumventing the dissolution in organic solvents, both natural-based and synthetic polymers with a high hydrosolubility are used to produce porous structures that are functionally robust and potentially noncytotoxic. Moreover, this technique was demonstrated to be effective in the fabrication of multi-layered scaffolds, which also exhibit widespread applications in biomimetic TE, i.e., scaffolds with a nano-scaled architecture. However, this technique presents the disadvantage of being significantly energy- and time-consuming, and the resulted pore sizes are substantially smaller than would be expected for supporting penetration and differentiation of certain types of cells [47,51,52].

## 3. Phase Separation

Phase separation is a widely explored scaffold fabrication technique with the advantage of tailoring mechanical properties and pore size of the foams produced for tissue engineering purposes [52]. A phase separation process takes advantage of the thermodynamic demixing of a homogeneous polymer–solvent (binary) or polymer–solvent–nonsolvent (ternary) solution to obtain a polymer-rich (with a high polymer concentration) and a polymer-lean phase (with a low polymer concentration) [53]. As a result, a solid matrix forms after the solidification of the polymer-rich phase, while the polymer-lean phase generates pores after solvent removal [54]. The phase separation can occur as a consequence of the diffusion between the solvent and a nonsolvent (immiscible or partially miscible solvent) or by reaching a demixing temperature [55]. The first process is called either diffusion induced phase separation (DIPS) or nonsolvent induced phase separation (NIPS), and the second one is known as thermally induced phase separation (TIPS). In the DIPS method, the first step is the molding of a thin layer of the polymer solution onto a solid support (i.e., tube, fibers), followed by immersion in a nonsolvent bath to induce the demixing [56]. The TIPS process consists of changes in temperature onto the homogenous polymer solution, creating two different phases in equilibrium [57]. Both processes are usually followed by extracting the obtained foams from the mold, a washing step in distilled water, and a desiccation stage to ensure the removal of any solvent trace [58,59,60].

### 3.1. DIPS

Using the DIPS method, scaffold morphology, mechanical properties, and separation performance are the result of the adoption of operating parameters during the process. Specifically, the solvent–nonsolvent nature and ratio, as well as the polymer type and concentration, must be chosen so that the polymer is easily dispersible in the chosen solvent, and the solvent and nonsolvent are mutually miscible [60]. Recently, Montesanto et al. also studied the effects of coagulation bath composition and desiccation environment to achieve better control on scaffold morphology [61]. They found that the morphology of the internal surface is more affected by the composition of the coagulation bath, whereas the desiccation environment affects the external surface of the produced scaffolds. Current researches on DIPS fabricated scaffolds focused on assessing biological response and mechanical performance of biopolymer based-scaffolds. Gangolli et al. [62] fabricated bilayered samples from PLGA and evaluated the indentation modulus and the odontoblastic differentiation of cultured human dental pulp stem cells (DPCSs). Utilizing DIPS, they manufactured two layers at different PLGA concentrations and observed a faster liquid-liquid phase separation at lower polymer concentration, along with a strict correlation between cell differentiation, stiffness, and topographical organization into scaffold morphology. In 2017, Rezabeigi et al. [63] fabricated a multifunctional bone scaffold made of bioactive glass (BG) and PLA through the DIPS technique. They found that the addition of the particles reduced the nonsolvent nature, hence leading to a more compact structure, with deformed macropores and lower porosity. Via the same route, Liu et al. [11] produced PCL/hydroxyapatite (HA) composite scaffolds for bone regeneration, with tailored macro/micro-porous structure, high mechanical properties, and excellent in vitro bioactivity using the NIPS-based 3D plotting technique. Specifically, microporous PCL/HA composite filaments were created using the exchange of the solvent and the nonsolvent during the deposition process. As a result, the control of the macro/micro-porous structure and the addition of HA improved the mechanical properties (i.e., ultimate tensile strength and compressive yield strength) and apatite-forming ability of the fabricated scaffolds.

Sohn et al. fabricated PCL dual-pore scaffolds with interconnected pores and used DIPS and Wire-Network Molding (WNM) techniques in the manufacturing process. They reported that structures fabricated using a combination of DIPS technique and WNM, which is a mold assembly method using a wire-cutting process, might be more advantageous compared to the conventionally manufactured DIPS structures due to a lower degree of scaffold collapsing during the fabrication process and higher number of attached Saos-2 cells [60]. Montesanto et al. [64] investigated the structural properties of PLLA membranes fabricated with a modified DIPS process, including the sequential immersion into two coagulation baths, and evaluated the formation of a functional lung epithelial barrier after 5 days of the NCl-H441 cells’ culture on the produced scaffolds. The results showed that the PLLA scaffolds examined had higher porosity and permeability compared to PLLA membranes produced using standard DIPS, hence enhancing epithelial cell function. Furthermore, the PLLA scaffolds also supported the formation of a lung epithelial barrier capable of responding to the induction of a proinflammatory stimulus.

**Figure 4:**
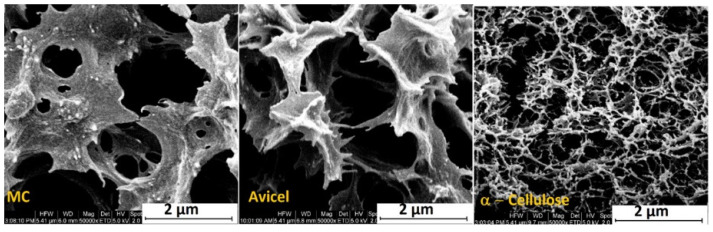
Influence of the cellulose type on the porous film structure (cross-section of 8 wt % cellulose-based films) [66]. Reprint with permission from ACS.

Because of their higher biodegradability and biocompatibility, natural polymer-based scaffolds were also investigated by DIPS technology. Kasoju et al. [65] produced silk fibroin hydrogels using the principle of nonsolvent induced phase separation to induce the gelation process. They found that the gelation time and the properties of the hydrogels were influenced by the amount of the nonsolvent, silk concentration, and incubation temperature. Recently, Wittmar et al. [66] studied for the first time the NIPS process starting from biopolymer solutions in ionic liquid-based solvents, with or without acetone as a co-solvent, to produce porous cellulose film structures. The results demonstrated that cellulose with a high polymerization degree generated more compact films under identical NIPS conditions, as Figure 4 shows. The authors considered the much higher viscosity of the high-polymerized cellulose as the main reason for the difference in the porous structure since this effect may prevent a fast exchange between the solvent and nonsolvent. By using the DIPS technique, the average size and the size distribution of the pores, as well as the dimension and shape of the scaffolds, can be easily tailored by choosing some fundamental process parameters [67]. Some limitations of this technique concern the evaporation-induced shrinkage of the produced scaffolds and the formation of macro voids that could weaken the mechanical properties of the constructs [67,68].

### 3.2. TIPS

**Figure 5:**
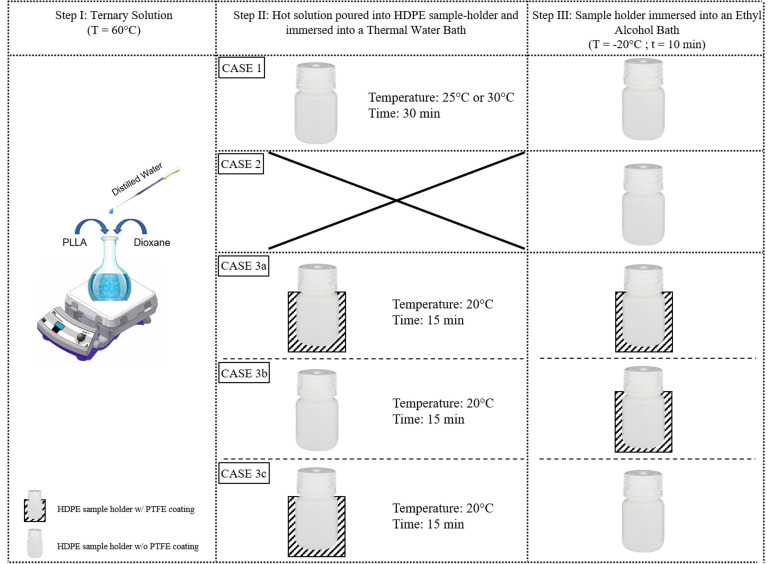
Schematic representation of scaffold preparation steps. A ternary solution (PLLA/dioxane/water) was prepared and kept at T = 60 °C, and hot poured into HDPE cylindrical sample-holders. Case 1: the sample holder was maintained at the demixing temperature of 25 °C (or 30 °C) for 30 min; thereafter, the system was suddenly quenched by pool immersion in an ethyl alcohol bath (EAB) at a temperature of −20 °C for at least 10 min to freeze the as-obtained structure. Case 2: the sample-holder was placed directly at −20 °C for 20 min being subjected to the so-called Direct Quench (DQ). Case 3 a,b,c: the sample holder was kept uncoated or embedded in a PTFE coating before immersion in the thermostatic water bath (TWB) and then maintained at the demixing temperature of 20 °C for 15 min; thereafter, the system was suddenly quenched by pool immersion in an ethyl alcohol bath (EAB) or at a temperature of −20 °C for at least 10 min to freeze the as-obtained structure [69].

In thermally induced phase separation, the porous scaffold morphology is significantly affected by the change in the parameters, such as types of polymer, polymer concentration, solvent/nonsolvent ratio, and thermal history (i.e., temperature versus time path) [4]. The fabrication of polymeric scaffold was carried out by Lombardo et al. [69] via TIPS by using PLLA to assess the morphology of scaffolds prepared by varying demixing temperatures, times, and immersion conditions, as shown in Figure 5. According to their findings, while keeping constant the cooling path, an increase of the demixing time determines larger pore dimensions. 

Moreover, as the polymer concentration increases, pore dimension and porosity decrease accordingly while obtaining a well-interconnected macroporous structure [18]. The mechanical performances and morphological properties have a significant relationship in the scaffold fabrication process. Luo et al. [70] produced scaffolds with different porosities and microstructures using TIPS to study the relationship between the polymer average molecular weight and tensile strength. In the study of Sabzi et al. [18], desirable mechanical properties and the control over fiber diameter and orientation of nanofibrous gelatin scaffolds were obtained by combining TIPS with the particulate leaching technique. Additionally, Zheng et al. [71] examined the combination of TIPS and particulate leaching to produce 3D nanofibrous scaffolds with well-defined macro and microstructures and the incorporation 5 wt % of 45S5 bioglass particles. They fabricated and characterized the as-obtained gelatin nanofibrous scaffolds in terms of mechanical properties and osteogenic differentiation. Remaining in the field bone regeneration, Farzamfar et al. [72] applied the TIPS technique to produce PCL/PLA scaffolds containing different levels of tetracycline hydrochloride (TCH) antibiotic for testing their local administration efficiency. The evaluation of the bone healing activity was confirmed by the osteoinductive properties of the produced scaffolds, as well as by the in vitro cell proliferation and viability. Finally, they found that the highest in vivo bone formation in a rat femoral defect occurred in scaffolds incorporating 10 wt % of TCH antibiotic. In another related study by Gupte et al. [73], the effects of experimental parameters on the pore size and BMSC differentiation of nanofibrous PLLA scaffolds for a combined TIPS/sugar porogen method was investigated. It was observed that chondrogenic differentiation and control of vascularization were strongly dependent on the manufacturing process, which, in its turn, is effective in modulating scaffold pore dimensions. Diaz et al. [74] also studied the influence of the use of particles and fabrication parameters of the TIPS process on the thermal and mechanical properties of poly(caprolactone; PCL)/nanohydroxyapatite (nHA)/multiwalled carbon nanotube (MWCNT) composite scaffolds. They found that it is possible to fabricate composite scaffold structures using nano carbons and hydroxyapatite via TIPS by a process optimization based on the application of ultrasonic dispersion. It was also shown that specimens prepared with no more than 10% nHA and 5% CNTs exhibit satisfactory in vitro degradation and mechanical properties for the design and fabrication of scaffolds with potential use in bone tissue regeneration. Microparticles and polymer matrix composites with bioactive ceramic phase have received much attention in the tissue engineering field thanks to their potential to enhance structural and biological performances while taking advantage of the well-acquainted polymers’ formability [75]. The use of TIPS-produced scaffolds with the incorporation of ceramic nanoparticles was recently studied by Carfì Pavia et al. [76,77]. The authors have conducted extensive research on the integration of TIPS and Poly-L-lactic acid (PLLA)/Hydroxyapatite (HA) to fabricate porous scaffolds applicable in bone tissue engineering. They reported the promising success of TIPS in ensuring the incorporation and spatially homogeneous distribution of HA particles in the polymer matrix, as confirmed by several characterization techniques. Salerno and Domingo [22] also fabricated composite scaffolds from either polycaprolactone (PCL) or PCL loaded with hydroxyapatite (HA) nanoparticles by TIPS. The effects of the integration of HA nanoparticles into the polymer and the freezing temperature on the scaffold structure were studied. On the other hand, the manufacture of composite scaffolds via TIPS was carried out by Erickson et al. [10] to produce multiphasic scaffolds by using chitosan, alginate, hyaluronic acid, and hydroxyapatite to enhance cell attachment and osteoconductivity in osteochondral tissue regeneration. According to their findings, the TIPS method is advantageous over previously reported techniques in creating well-integrated multi-layer scaffolds due to the tunability of the stiffness, and pore size of each layer, as well as the integration of bioactive factors to each specific region.

Overall, the TIPS technique is an effective conventional fabrication technology for tissue engineering scaffolds that allows one to generate porous matrices with an interconnected pore network while offering low production cost and relatively easy processability [78]. Moreover, it is one of the most effective methods for producing polymeric foams with porosity over 95% [79]. Despite the presence of organic solvents, a literature review confirms that the TIPS process ensures the achievement of a final structure without any remaining solvent trace, hence, preserving biocompatibility. However, this phase separation method suffers some disadvantages such as limited material selection and reduced reproducibility, as well as unspecific resolution [80].

## 4. Electrospinning

**Figure 6:**
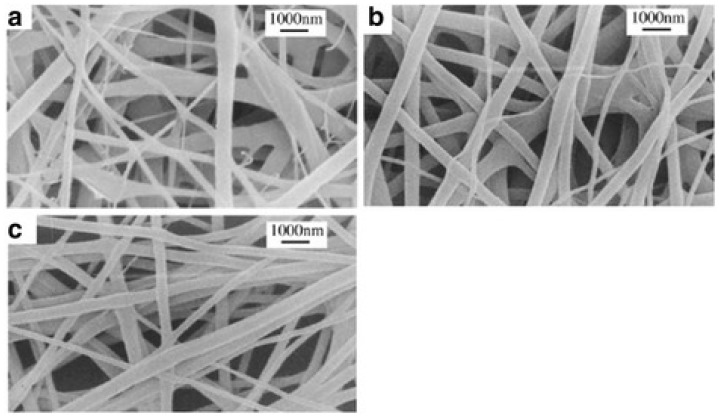
SEM micrographs of prepared scaffolds. (**a**) SF. (**b**) SF+rhBMP2. (**c**) 4SF+rhBMP2 [85]. Reprint with permission from PMC.

Electrospinning involves the production of continuous fibers from inducing the charged filaments of polymer solutions by using an electric force directed by a high-voltage system equipped with a syringe connected to a metallic needle, a pressure pump, and a grounded stationary or rotating collector [81,82,83]. The liquid solution, pumped into the syringe, flows up to onto a pendent droplet suspended at the needle tip, which is then stretched continuously to form a cone-like structure, called the Taylor cone, while increasing the applied voltage. Successive jet initiates from the tip of the Taylor cone, and then it is continuously stretched and thinned down by the electrostatic force overcoming the liquid surface tension [81]. The electrospinning process requires particular attention to factors influencing the structure and size of electrospun fibers and preventing the break-up of the fibers during the process, such as solution concentration, polymer average molecular weight and distribution, viscosity, electric field, solution feed rate, and voltage [84]. Several research groups have utilized the electrospinning process for natural polymers-based tissue engineering scaffolds. Recently, one research group explored this field using silk fibroin (SF) cross-linked via glutaraldehyde with osteoinductive recombinant human bone morphogenic protein-2 (rhBMP2) [85]. Their goal was to use electrospinning to fabricate osteoinductive scaffolds with appropriate mechanical properties for bone formation. Figure 6 shows an SEM of produced SF and SF+BMP2 scaffolds with uniform architecture in SF nanofibers and a non-uniformity in fiber diameter from BMP2 conjugation. 

**Figure 7:**
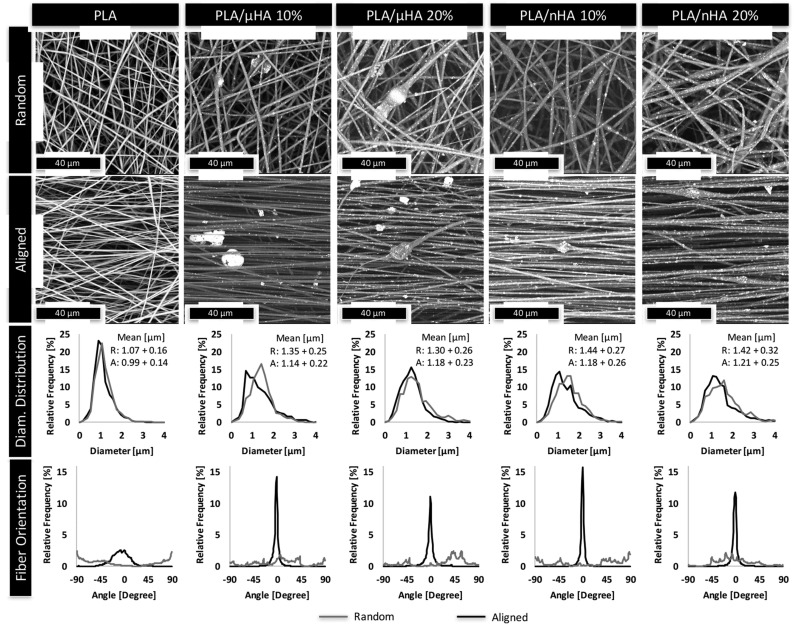
SEM micrographs of random and aligned electrospun mats at different µHA and nHA concentrations and their corresponding fiber diameter and fiber orientation distribution [92].

Another research group validated the combination of electrospinning and cross-linking to promote fiber stability and morphological maintenance of electrospun matrices of gelatin, which is a soluble protein derived from the partial hydrolysis of collagen [16]. The authors reported that the stabilization of ultrafine gelatin fibers via cross-linking can be achieved either by physical or chemical methods and that these methodologies can either be applied once completing the electrospinning process (i.e., post-processing cross-linking) or during the electrospinning process (i.e., in situ cross-linking). The use of decellularized extracellular matrix (dECM) scaffolds is a relatively new approach that aids in cell adhesion, proliferation, and differentiation. To obtain such a controlled and modulated architecture, electrospinning was investigated to reduce several limitations related to the size, shape, and physicochemical properties of dECM. Smoak et al. [86] evaluated the mechanical properties and degradation kinetics of fibrous scaffolds fabricated by electrospinning and muscle dECM. The authors pointed out that their electrospun scaffolds were fabricated without any carrier polymer and indicated that the resulting scaffolds were less soluble than other natural electrospun materials due to the limited solubility of muscle proteins. To improve the bioactivity of TE scaffolds, structures containing collagen and chitosan composites were fabricated by electrospinning with low amounts of PCL (about 5 vol %); this latter added to decrease the elevated viscosity of the initially prepared collagen/chitosan solution. By this procedure, more fluent processing was obtained, and scaffolds with more regular macroscopic and microscopic structures were successfully fabricated [87]. Within the context of producing biodegradable and biocompatible scaffolds with regular shape and smooth surface nanofibers, Sandri et al. [88] developed an electrospinning-based process and built up scaffolds entirely based on aqueous polysaccharide solutions. It was reported that the developed method and the use of Chitosan/Chondroitin sodium sulfate (CS) are promising in fabricating scaffolds allowing the skin healing process. For drug delivery testing purposes, Wongkanya et al. [89] manufactured Alginate/soy protein isolated nanofibers with antibacterial drug loading by using the electrospinning technique. The antibacterial activity of the resulting scaffolds was successfully assessed, along with their noncytotoxic behavior. The fibrous structure provided spaces for cell proliferation as well as controlled drug release. Most of the research in the area of TE scaffold fabrication using electrospinning is mainly focused on synthetic polymer materials that allow controllable degradation rate, porosity, and the ability to change shape and size [90]. However, some recent studies were performed to fabricate fibrous composite scaffolds including synthetic and natural polymers through electrospinning. A novel scaffold, specifically based on PVA-PCL combined with aloe vera, was produced as skin replacement due to its good hydrophilic properties, excellent cell viability, and promoted cell proliferation [91]. More recently, research groups focused on the mechanical properties and characterization of electrospun scaffolds derived from synthetic polymers. In the study of Lopresti et al. [92], micrometric and nanometric hydroxyapatite was incorporated in PLA electrospun scaffolds fabricated as aligned and randomly oriented fibers, as shown in Figure 7. Structural characterization and mechanical properties, including elastic modulus, tensile strength, and the elongation at break, were investigated. The authors concluded that the mechanical properties of the fabricated porous scaffolds were affected by HA concentration, leading to an increase in the elastic modulus of the nanofibers at higher HA percentage, with differences related to the filler dimension and the fiber orientation. Overall, the PLA/nHA 20% scaffold with the best elastic modulus improvement and homogeneous colonization by pre-osteoblastic cells resulted in a suitable candidate for bone tissue regeneration.

In another study by Lopresti et al. [93], a dual-jet electrospinning process was proposed to manufacture a series of PLA/PCL co-mingled nanofiber mats. The mechanical characterization and hydrolytic degradation of the resulted structures were assessed by using tensile tests and different buffered solutions (i.e., pH 4, pH 7, and pH 10), respectively. Their findings indicated that a dual-jet electrospinning system could produce a uniformly co-mingled structure of scaffolds by tuning the respective flow rates during the process. Han et al. [94] also employed electrospinning to fabricate fibrous PCL scaffolds with dimples on the surface with a hollow core. They utilized a co-axial nozzle to create the hollow fibers and developed microvasculature structures that degrade within a reasonable time frame for proper blood perfusion. The authors reported that the hollow structure expedited the degradation process, minimizing the undesired effects of remaining fibers after transplantation, and the higher surface roughness of dimpled fiber improved the maturity of endothelial cells and increased cell scaffold affinity. On the other hand, PCL scaffolds for vascular tissue engineering were fabricated as support for the creation of a polymer-cell complex in vitro with subsequent implantation in vivo. Dimopoulos et al. [95] verified the in vitro biocompatibility and cytotoxicity of electrospun PCL structures, as well as their significant effect on cellular behavior in vivo, i.e., their capability to induce cell attachment, migration, and differentiation. Additionally, the achieved fiber diameter, as well as mechanical properties of the produced scaffold, resulted comparable to the collagen fibers found in native vessels. Other research works that exploited the capabilities of electrospinning to fabricate scaffolds from polymers with thermosetting characteristic revealing manufacture complications include the study of Zhu et al. [94], where Poly(1,8-octanediol citrate) (POC)/PLLA fiber mats with improved mechanical properties and hydrophilicity were fabricated and the elastomer-based structures characterized via dynamic mechanical analysis, uniaxial tensile test, thermal analysis, in vitro degradation and biocompatibility test. 

The fabrication of nanofiber scaffold mainly occurs by the electrospinning method, which better supports a fine-tuning of surface area and aspect ratio in a fast and effective manner. Moreover, this technique allows the control over porosity, pore size, and diameter of fibers and a high degree of flexibility in creating the final form of scaffolds. Some disadvantages rely on the limited thickness of nanofibrous scaffolds produced by conventional electrospinning as well as on the difficulties in cell penetration inside the fibrous network [96,97].

## 5. Combination of Solution-Based Techniques

Solution-based technologies are widely used manufacturing methods to produce porous engineering scaffold [46]. Many works investigated the solution-based fabrication protocols to manufacture biodegradable polymeric scaffolds as this approach can create several solid forms starting from different materials, including thermoplastic polymers. Thermoplastic materials are utilized for melt molding and melt extrusion [98,99]. Frequently some additives, such as bioactive molecules or reinforcing fillers, can also be added to the polymers to obtain suitable mechanical properties and cellular response from the produced scaffolds [37,100]. Recently, diverse solution-based techniques have been combined to obtain a 3D porous structure with interconnected pores for TE applications. The TIPS technique is a commonly used method to fabricate TE scaffolds due to its simplicity and effectiveness in adjusting the structure and properties to the application of materials [70,74]. Carfì et al. [101] combined TIPS and DIPS to produce a multifunctional scaffold with easy-tunable pore size, porosity, and thickness. In their study, a double-structured PLLA scaffold was prepared by performing TIPS around a vessel-like scaffold fabricated by DIPS, resulting in an embedded structure for mimicking a vascularized tissue. This functionality was achieved thanks to the combination of the continuous porous morphology of the TIPS matrix with the in situ embedded DIPS-based scaffold; furthermore, no changes in the wall thickness of this latter were detected during the TIPS process [28]. Montanheiro et al. [102] employed the TIPS and freeze-drying techniques to produce poly(3-hydroxybutyrate-co-3-hydroxyvalerate) (PHBV) nanocomposite scaffolds reinforced with different amounts of cellulose nanocrystals (CNC). They obtained an adequate CNC dispersion, as well as a scaffold architecture with a combination of oriented pores, unidirectional channels, and some regions of random pores, allowing an efficient transport of cells and nutrients. As a result, reinforced scaffolds better supported mouse fibroblast cell attachment and proliferation, and showed an improved compression modulus with respect to neat PHBV scaffolds.

In other studies, TIPS was also combined with electrospinning, for instance, to generate a bilayer scaffold by electrospinning a synthetic polymer onto the outer surface of the TIPS-produced sponge inner layer [103]. However, in their electrospinning process, the produced grafts were unable to resist dilation by high pressures. On the other hand, Ngadiman et al. [104] developed a reinforced maghemite (γ-Fe2O3) scaffold by TIPS and electrospinning on a 3D-printed PVA template. As a result, the morphology and compressive strength of the obtained structure were suitable for hard tissue engineering scaffolds. Regarding the field of vascular tissue engineering, scaffolds with sufficient mechanical properties were fabricated by Guo et al. [105] by combining TIPS and electrospinning. They produced a bilayered tubular scaffold with an inner layer of a microporous GEL foam incorporating 10 wt % of salvianic acid (SA) and mesoporous silica nanoparticles (MSNs) by using the TIPS technique, whereas electrospun poly(ester-urethane)urea (PEEUU) nanofibers were deposited outside of the inner layer to strengthen the vascular scaffold. The final construct exhibited appropriate mechanical properties, along with a good proliferation and in vitro long-term anticoagulant efficacy of endothelial cells.

Samadian et al. [106] added Taurine (Tau) as a bioactive molecule to fabricate an architecture with positive biological activities and structural features for bone regeneration in electrospun gelatin nanofibers (GNFs) and TIP-based PLA/PCL scaffolds.

**Figure 8:**
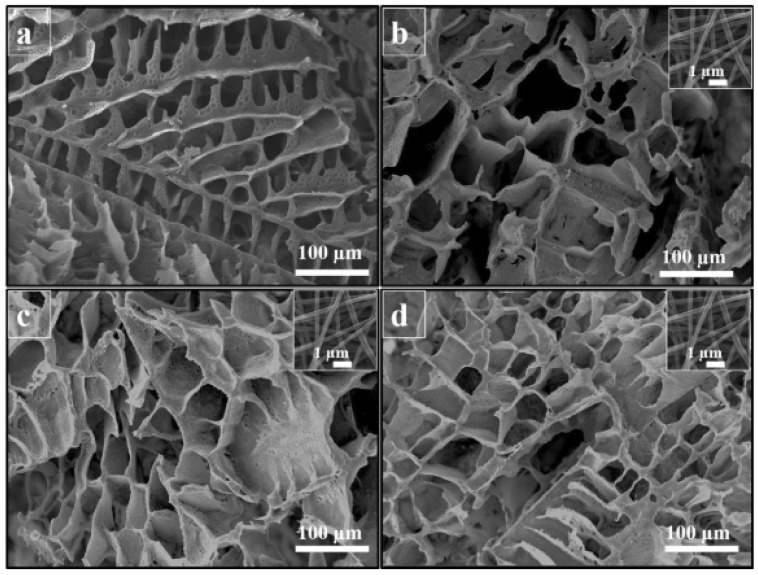
SEM micrograph of the prepared scaffolds. (**a**) PCL/PLA/GNF, (**b**) PCL/PLA/GNF/Tau 0.1%, (**c**) PCL/PLA/GNF/Tau 1%, and (**d**) PCL/PLA/GNF/Tau 10%. The insets show SEM micrograph of GNFs [106]. Reprint with permission from Springer Nature.

As shown in Figure 8, Tau was successfully incorporated into the scaffold structure and used as a bioactive molecule and physical additive that gradually increased hydrophilicity, biodegradability, and MG-63 cell proliferation. Then, scaffolds with highly positive effects on bone metabolism and regeneration were produced, as demonstrated by the tissue repair after 12 weeks post-implantation of the scaffolds.

**Figure 9:**
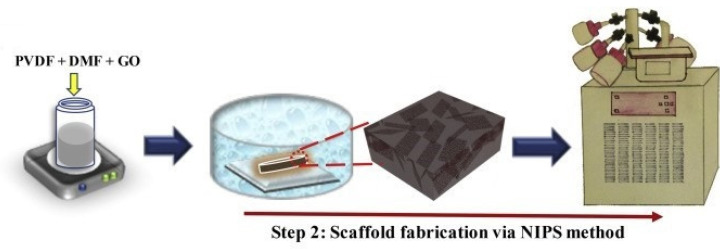
The schematic showing the immersion precipitation approach followed by a freeze-drying method to develop a porous PVDF-GO nanocomposite scaffold [108]. Reprint with permission from Elsevier.

Due to the possibility of avoiding the use of toxic organic solvents during scaffold preparation, freeze-drying was also used as a combined technique with electrospinning by Aghmiuni et al. to produce a substrate with improved physicochemical and biological functions for skin regeneration [26]. They also enhanced cell interactions and tissue remodeling by adopting hyaluronic acid (HA) as a signaling molecule maintaining the structural integrity of ECM in the scaffolds when interacting with proteins and proteoglycans. The addition of HA increased the water absorption and accelerated the degradation after 14 days. Cellular adhesion analysis indicated a facilitated loading of cells into scaffolds for cell attachment. Narouzi et al. [9] fabricated poly-caprolactone (PCL)/Gelatin (Gel) bilayer composite loaded with heparin, widely used as an anticoagulant drug, for vascular grafts via electrospinning and freeze-drying methods. The bilayer grafts displayed a connected pores network with mechanical properties similar to the coronary artery. Furthermore, the loading of heparin improved the endothelial cell attachment and decreased the risk of thrombosis. Besides electrospinning, other techniques, such as TIPS and DIPS, were also used with freeze-drying as hybrid fabrication technologies for TE scaffolds. Geng et al. [107] developed a 3D-printed biodegradable poly (glycerol-co-sebacic acid-co-l-lactic acid-co-polyethylene glycol) (PGSLP)-based scaffold that was internally filled with gelatin nanofibers. They fabricated the nanofibrous structured gelatin/PGSLP (NGP) scaffold employing a thermally induced phase separation (TIPS) technique, and then the macroporous structured gelatin/PGSLP (MGP) scaffold was prepared by directly freeze-drying. Due to the addition of gelatin, the local release of deferoxamine (DFO), which is essential for angiogenesis and osteogenesis in bone regeneration, was promoted. As a result, during the in vitro experiments, the mineralized nodule formation and osteogenic-related gene expression of bone marrow-derived mesenchymal stem cells (BMSCs) were achieved. In the end, the in vivo osteogenesis and the vascular formation were significantly promoted in scaffolds with DFO loading. In Kang’s study, highly porous poly(3-hydroxybutyrate) (PHB) scaffolds were fabricated using nonsolvent-induced phase separation followed by freeze-drying under 30 mTorr at −130 °C for 2 days [21]. The scaffolds showed well-connected micropores with increasing nonsolvent content and porosity in the range of 97 to 99%. This approach demonstrated to have a great potential in manufacturing a porous scaffold with proper morphology and mechanical properties due to the excellent control of the scaffold structure by NIPS. Cell proliferation on PHB scaffolds with various morphology and mechanical properties were then compared. For instance, microporosity was found to exert the largest impact on HaCaT cell proliferation on PHB scaffolds. Abzan et al. [108] recently prepared porous scaffolds by combining DIPS and freeze-drying as a simple and fast technique to fabricate polyvinylidene fluoride (PVDF) scaffolds with different concentrations of graphene oxide (GO) for nerve tissue engineering, as shown in Figure 9. Changes in the pore morphology from spherical to planar were observed in cross-section SEM images owing to the presence of GO nanosheets in the pore walls, and a uniformly porous surface was revealed only at lower GO contents.

Polyvinylidene fluoride (PVDF) scaffolds were also produced with relatively small pore size and uniform foam-like structure by the combination of NIPS, TIPS, and freeze-drying processes [109]. In this study, the effects of different working parameters, such as coagulation bath composition and temperature, soaking time, and solvent composition, were evaluated based on the physical, mechanical, and biological properties of porous PVDF scaffolds. By looking at the results, parameters promoting an enhanced crystallinity and β phase fraction allowed a significantly higher toughness and promoted cell spreading and proliferation.

## 6. Summary

This review describes recent signs of progress in the fabrication of scaffolds by solution-based techniques, including freeze-drying, phase separation, electrospinning, and a combination of these manufacturing methods. Several studies demonstrated the potential use of the produced scaffolds for clinical applications due to their tunable properties, uniformity in the porous 3D structures, interconnected pores, and appropriate mechanical properties. Due to the possibility of producing composite structures with different compositions over a wide range of additive materials, solution-based techniques are an efficient approach to fabricate scaffolds with suitable chemical, physical and biological characteristics for the desired regenerating tissue. Although porous scaffolds have a controlled pore size, constructs with high reproducibility in the spatial distribution of pore geometry cannot be precisely obtained using these manual-based techniques. Another limitation is the frequent use of organic/toxic solvents to dissolve polymers and other chemicals that may cause inflammatory responses. Recently, some progress was achieved to overcome these issues by modifying process conditions and/or to set up inorganic solutions and obtain nontoxic, fully formed scaffolds. A combination of solution-based techniques was also developed to produce high porosity structures without a negative impact on mechanical properties, such as compressive and tensile strength. By controlling the composition of the solution, the choice of process setups or post-processing treatment constructs with controlled biodegradability or bioresorbability can be fabricated so that tissue will eventually replace the scaffold. However, some limitations to address complex geometries within a single scaffold are currently studied in order to achieve the potential for clinical use. By developing novel mold shapes and combining different techniques, a scaffold with a large range of geometries can be achieved while maintaining homogeneous and regular characteristics for the repair of large defects.

Studies have shown that solution-based techniques can produce functional scaffolds as supports for the differentiation and proliferation of a large number of cell types. However, the fabrication of functional tissues and organs for in vivo testing at clinically relevant levels should be improved further.

In the future, a more extensive combination of techniques/biomaterials should be developed to overcome the observed limits in the customization of the size and shape of the bioengineered tissue. By improving traditional fabrication techniques, desired properties, such as uniform and interconnected pore structure, tunable degradation rate, and proper mechanical properties of scaffolds, should be reached simultaneously from a single manufacturing formulation. Freeze-drying, phase separation, and electrospinning can produce scaffolds with different structural morphologies without the requirement of expensive instruments. Therefore, a smart combination of these fabrication methods could successfully lead to the development of multi-layered matrices capable of mimicking the unique architecture of tissues and organs that are intrinsically difficult to design.

## Data Availability

Not applicable.

## References

[1] Scaffaro R., Lopresti F., Botta L., Rigogliuso S., Ghersi G. (2016). Integration of PCL and PLA in a monolithic porous scaffold for interface tissue engineering. J. Mech. Behav. Biomed. Mater..

[2] Donate R., Monzón M., Alemán-Domínguez M.E. (2020). Additive manufacturing of PLA-based scaffolds intended for bone regeneration and strategies to improve their biological properties. E-Polymers.

[3] Courtenay J., Sharma R., Scott J. (2018). Recent Advances in Modified Cellulose for Tissue Culture Applications. Molecules.

[4] Yang S., Leong K.-F.E., Du Z.M.E., Chua C.-K. (2001). The Design of Scaffolds for Use in Tissue Engineering. Part I. Traditional Factors. Tissue Eng..

[5] Eltom A., Zhong G., Muhammad A. (2019). Scaffold Techniques and Designs in Tissue Engineering Functions and Purposes: A Review. Adv. Mater. Sci. Eng..

[6] Deb P., Deoghare A.B., Borah A., Barua E., Das Lala S. (2018). Scaffold Development Using Biomaterials: A Review. Mater. Today Proc..

[7] Gupta S., Bissoyi A., Bit A. (2018). A Review on 3D Printable Techniques for Tissue Engineering. Bionanoscience.

[8] Bisht B., Hope A., Mukherjee A., Paul M.K. (2021). Advances in the Fabrication of Scaffold and 3D Printing of Biomimetic Bone Graft. Ann. Biomed. Eng..

[9] Norouzi S.K., Shamloo A. (2019). Bilayered heparinized vascular graft fabricated by combining electrospinning and freeze drying methods. Mater. Sci. Eng. C.

[10] Erickson A.E., Sun J., Lan Levengood S.K., Swanson S., Chang F.C., Tsao C.T., Zhang M. (2019). Chitosan-based composite bilayer scaffold as an in vitro osteochondral defect regeneration model. Biomed. Microdevices.

[11] Liu S., Zheng Y., Liu R., Tian C. (2020). Preparation and characterization of a novel polylactic acid/hydroxyapatite composite scaffold with biomimetic micro-nanofibrous porous structure. J. Mater. Sci. Mater. Med..

[12] Guo R., Chen S., Xiao X. (2019). Fabrication and characterization of poly (ethylenimine) modified poly (l-lactic acid) nanofibrous scaffolds. J. Biomater. Sci. Polym. Ed..

[13] Salehi M., Bastami F., Rezai Rad M., Nokhbatolfoghahaei H., Paknejad Z., Nazeman P., Hassani A., Khojasteh A. (2021). Investigation of cell-free poly lactic acid/nanoclay scaffolds prepared via thermally induced phase separation technique containing hydroxyapatite nanocarriers of erythropoietin for bone tissue engineering applications. Polym. Adv. Technol..

[14] McKenna E., Klein T.J., Doran M.R., Futrega K. (2020). Integration of an ultra-strong poly(lactic-co-glycolic acid) (PLGA) knitted mesh into a thermally induced phase separation (TIPS) PLGA porous structure to yield a thin biphasic scaffold suitable for dermal tissue engineering. Biofabrication.

[15] de Souza L., Alavarse A.C., da Vinci M.A., Bonvent J.J. (2021). The Synergistic Effect of Polymer Composition, Solvent Volatility, and Collector Distance on Pullulan and PVA Fiber Production by Rotary Jet Spinning. Fibers Polym..

[16] Campiglio C.E., Contessi Negrini N., Farè S., Draghi L. (2019). Cross-Linking Strategies for Electrospun Gelatin Scaffolds. Materials.

[17] Chen L., Wu Z., Zhou Y., Li L., Wang Y., Wang Z., Chen Y., Zhang P. (2017). Biomimetic porous collagen/hydroxyapatite scaffold for bone tissue engineering. J. Appl. Polym. Sci..

[18] Sabzi E., Abbasi F., Ghaleh H. (2020). Interconnected porous nanofibrous gelatin scaffolds prepared via a combined thermally induced phase separation/particulate leaching method. J. Biomater. Sci. Polym. Ed..

[19] Andrieux S., Drenckhan W., Stubenrauch C. (2017). Highly ordered biobased scaffolds: From liquid to solid foams. Polymer.

[20] Ghafari R., Jonoobi M., Amirabad L.M., Oksman K., Taheri A.R. (2019). Fabrication and characterization of novel bilayer scaffold from nanocellulose based aerogel for skin tissue engineering applications. Int. J. Biol. Macromol..

[21] Kang J., Hwang J.Y., Huh M., Yun S.I. (2020). Porous Poly(3-hydroxybutyrate) Scaffolds Prepared by Non-Solvent-Induced Phase Separation for Tissue Engineering. Macromol. Res..

[22] Salerno A., Domingo C. (2015). Pore structure properties of scaffolds constituted by aggregated microparticles of PCL and PCL-HA processed by phase separation. J. Porous Mater..

[23] Nune S.K., Rama K.S., Dirisala V.R., Chavali M.Y. (2017). Electrospinning of collagen nanofiber scaffolds for tissue repair and regeneration. Nanostructures for Novel Therapy: Synthesis, Characterization and Applications.

[24] Lopresti F., Keraite I., Ongaro A.E., Howarth N.M., Carrubba V.L., Kersaudy-Kerhoas M. (2020). Engineered membranes for residual cell trapping on microfluidic blood plasma separation systems: A comparison between porous and nanofibrous membranes. bioRxiv.

[25] Miszuk J.M., Hu J., Sun H. (2020). Biomimetic Nanofibrous 3D Materials for Craniofacial Bone Tissue Engineering. ACS Appl. Bio Mater..

[26] Izadyari Aghmiuni A., Heidari Keshel S., Sefat F., Akbarzadeh Khiyavi A. (2021). Fabrication of 3D hybrid scaffold by combination technique of electrospinning-like and freeze-drying to create mechanotransduction signals and mimic extracellular matrix function of skin. Mater. Sci. Eng. C.

[27] Sin D., Fitzpatrick J., Luckman P., Wolvetang E.J., Cooper-White J.J. (2012). Fabrication of nanopatterned, porous microspheres using a glass capillary microfluidic device. Soft Matter.

[28] Gao J., Chung T.S. (2021). Membranes made from nonsolvent-thermally induced phase separation (N-TIPS) for decellularization of blood in dry plasma spot (DPS) applications. Chem. Eng. Sci..

[29] Li Y., Yang S.T. (2001). Effects of three-dimensional scaffolds on cell organization and tissue development. Biotechnol. Bioprocess Eng..

[30] Fu N., Zhang X., Sui L., Liu M., Lin Y. (2017). Application of Scaffold Materials in Cartilage Tissue Engineering. Cartilage Regeneration, Stem Cell Biology and Regenerative Medicine.

[31] Keeney M., Pandit A. (2009). The osteochondral junction and its repair via bi-phasic tissue engineering scaffolds. Tissue Eng. Part B Rev..

[32] Oh S.H., Kim T.H., Im G., Lee J.H. (2010). Investigation of pore size effect on chondrogenic differentiation of adipose stem cells using a pore size gradient scaffold. Biomacromolecules.

[33] Eberli D. (2011). Tissue Engineering for Tissue and Organ Regeneration.

[34] Vacanti J.P., Langer R. (1999). Tissue engineering: The design and fabrication of living replacement devices for surgical reconstruction and transplantation. Lancet.

[35] Serbo J.V., Gerecht S. (2013). Vascular tissue engineering: Biodegradable scaffold platforms to promote angiogenesis. Stem Cell Res. Ther..

[36] Ou S.-F., Tsao Y.-L., Lin W.-C., Wang Y.-T., Wang L., Fan F.-Y. (2020). Novel Epigallocatechin-3-Gallate (EGCG)-Loaded Mesoporous Bioglass Scaffolds for Bone Recruitment Applications. Appl. Sci..

[37] Liang C., Luo Y., Yang G., Xia D., Liu L., Zhang X., Wang H. (2018). Graphene Oxide Hybridized nHAC/PLGA Scaffolds Facilitate the Proliferation of MC3T3-E1 Cells. Nanoscale Res. Lett..

[38] Siddiqui N., Asawa S., Birru B., Baadhe R., Rao S. (2018). PCL-Based Composite Scaffold Matrices for Tissue Engineering Applications. Mol. Biotechnol..

[39] Fereshteh Z. (2018). Freeze-drying technologies for 3D scaffold engineering. Functional 3D Tissue Engineering Scaffolds: Materials, Technologies, and Applications.

[40] Ranganathan N., Mugeshwaran A., Bensingh R.J., Kader M.A., Nayak S.K. (2019). Biopolymeric scaffolds for tissue engineering application. Biomedical Engineering and Its Applications in Healthcare.

[41] Brougham C.M., Levingstone T.J., Shen N., Cooney G.M., Jockenhoevel S., Flanagan T.C., O’Brien F.J. (2017). Freeze-Drying as a Novel Biofabrication Method for Achieving a Controlled Microarchitecture within Large, Complex Natural Biomaterial Scaffolds. Adv. Healthc. Mater..

[42] Grenier J., Duval H., Barou F., Lv P., David B., Letourneur D. (2019). Mechanisms of pore formation in hydrogel scaffolds textured by freeze-drying. Acta Biomater..

[43] Valencia C., Valencia C., Zuluaga F., Valencia M., Mina J., Grande-Tovar C. (2018). Synthesis and Application of Scaffolds of Chitosan-Graphene Oxide by the Freeze-Drying Method for Tissue Regeneration. Molecules.

[44] Mesgar A.S., Mohammadi Z., Khosrovan S. (2018). Improvement of mechanical properties and in vitro bioactivity of freeze-dried gelatin/chitosan scaffolds by functionalized carbon nanotubes. Int. J. Polym. Mater. Polym. Biomater..

[45] Zhai C., Fei H., Hu J., Wang Z., Xu S., Zuo Q., Li Z., Wang Z., Liang W., Fan W. (2018). Repair of Articular Osteochondral Defects Using an Integrated and Biomimetic Trilayered Scaffold. Tissue Eng. Part A.

[46] Patel H., Bonde M., Srinivasan G. (2011). Biodegradable polymer scaffold for tissue engineering. Trends Biomater. Artif. Organs.

[47] Dattola E., Parrotta E.I., Scalise S., Perozziello G., Limongi T., Candeloro P., Coluccio M.L., Maletta C., Bruno L., De Angelis M.T. (2019). Development of 3D PVA scaffolds for cardiac tissue engineering and cell screening applications. RSC Adv..

[48] Mozafari M., Kargozar S., de Santiago G.T., Mohammadi M.R., Milan P.B., Foroutan Koudehi M., Aghabarari B., Nourani M.R. (2018). Synthesis and characterisation of highly interconnected porous poly(ε-caprolactone)-collagen scaffolds: A therapeutic design to facilitate tendon regeneration. Mater. Technol..

[49] Namini M.S., Bayat N., Tajerian R., Ebrahimi-Barough S., Azami M., Irani S., Jangjoo S., Shirian S., Ai J. (2018). A comparison study on the behavior of human endometrial stem cell-derived osteoblast cells on PLGA/HA nanocomposite scaffolds fabricated by electrospinning and freeze-drying methods. J. Orthop. Surg. Res..

[50] Haugh M.G., Murphy C.M., O’Brien F.J. (2010). Novel freeze-drying methods to produce a range of collagen- glycosaminoglycan scaffolds with tailored mean pore sizes. Tissue Eng. Part C Methods.

[51] Izquierdo-Barba I. (2014). Scaffold Designing. Bio-Ceramics with Clinical Applications.

[52] Liu S., Zheng Y., Hu J., Wu Z., Chen H. (2020). Fabrication and characterization of polylactic acid/polycaprolactone composite macroporous micro-nanofiber scaffolds by phase separation. New J. Chem..

[53] Zeng S., Cui Z., Yang Z., Si J., Wang Q., Wang X., Peng K., Chen W. (2016). Characterization of highly interconnected porous poly(lactic acid) and chitosan-coated poly(lactic acid) scaffold fabricated by vacuum-assisted resin transfer molding and particle leaching. J. Mater. Sci..

[54] Lu T., Li Y., Chen T. (2013). Techniques for fabrication and construction of three-dimensional scaffolds for tissue engineering. Int. J. Nanomed..

[55] Wang L., Abedalwafa M., Wang F., Li C. (2013). Biodegradable Poly-Epsilon-Caprolactone (PCL) for tissue Engineering Applications: A Review. Rev. Adv. Mater. Sci.

[56] la Carrubba V., Pavia F.C., Brucato V. (2010). Tubular scaffold for vascular tissue engineering application. Int. J. Mater. Form..

[57] Geven M.A., Lapomarda A., Guillaume O., Sprecher C.M., Eglin D., Vozzi G., Grijpma D.W. (2021). Osteogenic differentiation of hBMSCs on porous photo-crosslinked poly(trimethylene carbonate) and nano-hydroxyapatite composites. Eur. Polym. J..

[58] Conoscenti G., Schneider T., Stoelzel K., Carfì Pavia F., Brucato V., Goegele C., La Carrubba V., Schulze-Tanzil G. (2017). PLLA scaffolds produced by thermally induced phase separation (TIPS) allow human chondrocyte growth and extracellular matrix formation dependent on pore size. Mater. Sci. Eng. C.

[59] Thavornyutikarn B., Chantarapanich N., Sitthiseripratip K., Thouas G.A., Chen Q. (2014). Bone tissue engineering scaffolding: Computer-aided scaffolding techniques. Prog. Biomater..

[60] Guillen G.R., Pan Y., Li M., Hoek E.M.V. (2011). Preparation and characterization of membranes formed by nonsolvent induced phase separation: A review. Ind. Eng. Chem. Res..

[61] Montesanto S., Mannella G.A., Carfì Pavia F., La Carrubba V., Brucato V. (2015). Coagulation bath composition and desiccation environment as tuning parameters to prepare skinless membranes via diffusion induced phase separation. J. Appl. Polym. Sci..

[62] Gangolli R.A., Devlin S.M., Gerstenhaber J.A., Lelkes P.I., Yang M. (2019). A Bilayered Poly (Lactic-Co-Glycolic Acid) Scaffold Provides Differential Cues for the Differentiation of Dental Pulp Stem Cells. Tissue Eng. Part A.

[63] Rezabeigi E., Wood-Adams P.M., Drew R.A.L. (2017). Morphological examination of highly porous polylactic acid/Bioglass scaffolds produced via nonsolvent induced phase separation. J. Biomed. Mater. Res. Part B Appl. Biomater..

[64] Montesanto S., Smithers N.P., Bucchieri F., Brucato V., Carrubba V.L., Davies D.E., Conforti F. (2019). Establishment of a pulmonary epithelial barrier on biodegradable poly-L-lactic-acid membranes. PLoS ONE.

[65] Kasoju N., Hawkins N., Pop-Georgievski O., Kubies D., Vollrath F. (2016). Silk fibroin gelation via non-solvent induced phase separation. Biomater. Sci..

[66] Wittmar A.S.M., Koch D., Prymak O., Ulbricht M. (2020). Factors Affecting the Nonsolvent-Induced Phase Separation of Cellulose from Ionic Liquid-Based Solutions. ACS Omega.

[67] Figoli A., De Luca G., Longavita E., Drioli E. (2007). PEEKWC Capsules Prepared by Phase Inversion Technique: A Morphological and Dimensional Study. Sep. Sci. Technol..

[68] Jung J.T., Kim J.F., Wang H.H., Di Nicolo E., Drioli E., Lee Y.M. (2016). Understanding the non-solvent induced phase separation (NIPS) effect during the fabrication of microporous PVDF membranes via thermally induced phase separation (TIPS). J. Memb. Sci..

[69] Lombardo M.E., Carfì Pavia F., Vitrano I., Ghersi G., Brucato V., Rosei F., La Carrubba V. (2019). PLLA scaffolds with controlled architecture as potential microenvironment for in vitro tumor model. Tissue Cell.

[70] Luo Y., Yang F., Li C., Wang F., Zhu H., Guo Y. (2021). Effect of the molecular weight of polymer and diluent on the performance of hydrophilic poly(vinyl butyral) porous heddle via thermally induced phase separation. Mater. Chem. Phys..

[71] Zheng X., Liu Y., Liu Y., Pan Y., Yao Q. (2021). Novel three-dimensional bioglass functionalized gelatin nanofibrous scaffolds for bone regeneration. J. Biomed. Mater. Res. Part B Appl. Biomater..

[72] Farzamfar S., Naseri-Nosar M., Sahrapeyma H., Ehterami A., Goodarzi A., Rahmati M., Ahmadi Lakalayeh G., Ghorbani S., Vaez A., Salehi M. (2019). Tetracycline hydrochloride-containing poly (ε-caprolactone)/poly lactic acid scaffold for bone tissue engineering application: In vitro and in vivo study. Int. J. Polym. Mater. Polym. Biomater..

[73] Gupte M.J., Swanson W.B., Hu J., Jin X., Ma H., Zhang Z., Liu Z., Feng K., Feng G., Xiao G. (2018). Pore Size Directs Bone Marrow Stromal Cell Fate and Tissue Regeneration in Nanofibrous Macroporous Scaffolds by Mediating Vascularization. Acta Biomater..

[74] Díaz E., Aresti J., León J. (2020). Evaluation of physicochemical and mechanical properties with the in vitro degradation of PCL/nHA/MWCNT composite scaffolds. J. Reinf. Plast. Compos..

[75] Heo S.-J., Kim S.-E., Wei J., Hyun Y.-T., Yun H.-S., Kim D.-H., Shin J.W., Shin J.-W. (2008). Fabrication and characterization of novel nano- and micro-HA/PCL composite scaffolds using a modified rapid prototyping process. J. Biomed. Mater. Res. Part A.

[76] Carfì Pavia F., Conoscenti G., Greco S., La Carrubba V., Ghersi G., Brucato V. (2018). Preparation, characterization and in vitro test of composites poly-lactic acid/hydroxyapatite scaffolds for bone tissue engineering. Int. J. Biol. Macromol..

[77] Carfì Pavia F., Palumbo F.S., La Carrubba V., Bongiovì F., Brucato V., Pitarresi G., Giammona G. (2016). Modulation of physical and biological properties of a composite PLLA and polyaspartamide derivative obtained via thermally induced phase separation (TIPS) technique. Mater. Sci. Eng. C.

[78] Zeinali R., Del Valle L.J., Torras J., Puiggalí J. (2021). Recent progress on biodegradable tissue engineering scaffolds prepared by thermally-induced phase separation (Tips). Int. J. Mol. Sci..

[79] Lou T., Wang X., Yan X., Miao Y., Long Y.Z., Yin H.L., Sun B., Song G. (2016). Fabrication and biocompatibility of poly(l-lactic acid) and chitosan composite scaffolds with hierarchical microstructures. Mater. Sci. Eng. C.

[80] Nikolova M.P., Chavali M.S. (2019). Recent advances in biomaterials for 3D scaffolds: A review. Bioact. Mater..

[81] Das A., Balakrishnan N.T.M., Joyner J.D., Medhavi N., Manaf O., Jabeen Fatima M.J., Ahn J.-H., Ali W., Prasanth R. (2021). Electrospinning: The State of Art Technique for the Production of Nanofibers and Nanofibrous Membranes for Advanced Engineering Applications.

[82] Lopresti F., Pavia F.C., Ceraulo M., Capuana E., Brucato V., Ghersi G., Botta L., La Carrubba V. (2021). Physical and biological properties of electrospun poly(d,l-lactide)/nanoclay and poly(d,l-lactide)/nanosilica nanofibrous scaffold for bone tissue engineering. J. Biomed. Mater. Res. Part A.

[83] Wang C., Wang M. (2014). Electrospun multifunctional tissue engineering scaffolds. Front. Mater. Sci..

[84] Koyyada A., Orsu P. (2020). Recent Advancements and Associated Challenges of Scaffold Fabrication Techniques in Tissue Engineering Applications. Regen. Eng. Transl. Med..

[85] Du G.Y., He S.W., Sun C.X., Mi L.D. (2017). Bone Morphogenic Protein-2 (rhBMP2)-Loaded Silk Fibroin Scaffolds to Enhance the Osteoinductivity in Bone Tissue Engineering. Nanoscale Res. Lett..

[86] Smoak M.M., Han A., Watson E., Kishan A., Grande-Allen K.J., Cosgriff-Hernandez E., Mikos A.G. (2019). Fabrication and characterization of electrospun decellularized muscle-derived scaffolds. Tissue Eng. Part C Methods.

[87] Guo S., He L., Yang R., Chen B., Xie X., Jiang B., Weidong T., Ding Y. (2020). Enhanced effects of electrospun collagen-chitosan nanofiber membranes on guided bone regeneration. J. Biomater. Sci. Polym. Ed..

[88] Sandri G., Rossi S., Bonferoni M.C., Miele D., Faccendini A., Del Favero E., Di Cola E., Icaro Cornaglia A., Boselli C., Luxbacher T. (2019). Chitosan/glycosaminoglycan scaffolds for skin reparation. Carbohydr. Polym..

[89] Wongkanya R., Chuysinuan P., Pengsuk C., Techasakul S., Lirdprapamongkol K., Svasti J., Nooeaid P. (2017). Electrospinning of alginate/soy protein isolated nanofibers and their release characteristics for biomedical applications. J. Sci. Adv. Mater. Devices.

[90] Salah N.A. (2017). In Vitro Bronchial Mucosa Model Using Air-Liquid Interface Culture on PLLA Electrospun Membrane.

[91] Shabannejad M., Nourbakhsh M.S., Salati A., Bahrami Z. (2020). Fabrication and Characterization of Electrospun Scaffold Based on Polycaprolactone-Aloe vera and Polyvinyl Alcohol for Skin Tissue Engineering. Fibers Polym..

[92] Lopresti F., Carfì Pavia F., Vitrano I., Kersaudy-Kerhoas M., Brucato V., La Carrubba V. (2020). Effect of hydroxyapatite concentration and size on morpho-mechanical properties of PLA-based randomly oriented and aligned electrospun nanofibrous mats. J. Mech. Behav. Biomed. Mater..

[93] Scaffaro R., Lopresti F., Botta L. (2017). Preparation, characterization and hydrolytic degradation of PLA/PCL co-mingled nanofibrous mats prepared via dual-jet electrospinning. Eur. Polym. J..

[94] Han J.H., Ko U.H., Kim H.J., Kim S., Jeon J.S., Shin J.H. (2021). Electrospun Microvasculature for Rapid Vascular Network Restoration. Tissue Eng. Regen. Med..

[95] Dimopoulos A., Markatos D.N., Mitropoulou A., Panagiotopoulos I., Koletsis E., Mavrilas D. (2021). A novel polymeric fibrous microstructured biodegradable small-caliber tubular scaffold for cardiovascular tissue engineering. J. Mater. Sci. Mater. Med..

[96] Christy P.N., Basha S.K., Kumari V.S., Bashir A.K.H., Maaza M., Kaviyarasu K., Arasu M.V., Al-Dhabi N.A., Ignacimuthu S. (2020). Biopolymeric nanocomposite scaffolds for bone tissue engineering applications—A review. J. Drug Deliv. Sci. Technol..

[97] Tan G.Z., Zhou Y. (2019). Electrospinning of biomimetic fibrous scaffolds for tissue engineering: A review. Int. J. Polym. Mater. Polym. Biomater..

[98] Calore A.R., Sinha R., Harings J., Bernaerts K.V., Mota C., Moroni L. (2021). Additive Manufacturing Using Melt Extruded Thermoplastics for Tissue Engineering. Methods in Molecular Biology.

[99] Gorth D., Webster T.J. (2010). Matrices for tissue engineering and regenerative medicine. Biomaterials for Artificial Organs.

[100] Dabouian A., Bakhshi H., Irani S., Pezeshki-Modaress M. (2018). β-Carotene: A natural osteogen to fabricate osteoinductive electrospun scaffolds. RSC Adv..

[101] Carfì Pavia F., La Carrubba V., Ghersi G., Greco S., Brucato V. (2017). Double flow bioreactor for in vitro test of drug delivery. Curr. Drug Deliv..

[102] Montanheiro T.L.d.A., Montagna L.S., Patrulea V., Jordan O., Borchard G., Lobato G.M.M., Catalani L.H., Lemes A.P. (2019). Evaluation of cellulose nanocrystal addition on morphology, compression modulus and cytotoxicity of poly(3-hydroxybutyrate-co-3-hydroxyvalerate) scaffolds. J. Mater. Sci..

[103] Matsuzaki Y., Iwaki R., Reinhardt J.W., Chang Y.C., Miyamoto S., Kelly J., Zbinden J., Blum K., Mirhaidari G., Ulziibayar A. (2020). The effect of pore diameter on neo-tissue formation in electrospun biodegradable tissue-engineered arterial grafts in a large animal model. Acta Biomater..

[104] Ngadiman N., Yusof N., Idris A., Fallahiarezoudar E., Kurniawan D. (2018). Novel Processing Technique to Produce Three Dimensional Polyvinyl Alcohol/Maghemite Nanofiber Scaffold Suitable for Hard Tissues. Polymers.

[105] Guo X., Zhu J., Zhang H., You Z., Morsi Y., Mo X., Zhu T. (2019). Facile preparation of a controlled-release tubular scaffold for blood vessel implantation. J. Colloid Interface Sci..

[106] Samadian H., Farzamfar S., Vaez A., Ehterami A., Bit A., Alam M., Goodarzi A., Darya G., Salehi M. (2020). A tailored polylactic acid/polycaprolactone biodegradable and bioactive 3D porous scaffold containing gelatin nanofibers and Taurine for bone regeneration. Sci. Rep..

[107] Geng M., Zhang Q., Gu J., Yang J., Du H., Jia Y., Zhou X., He C. (2021). Construction of a nanofiber network within 3D printed scaffolds for vascularized bone regeneration. Biomater. Sci..

[108] Abzan N., Kharaziha M., Labbaf S. (2019). Development of three-dimensional piezoelectric polyvinylidene fluoride-graphene oxide scaffold by non-solvent induced phase separation method for nerve tissue engineering. Mater. Des..

[109] Holland T.A., Mikos A.G. (2006). Review: Biodegradable polymeric scaffolds. Improvements in bone tissue engineering through controlled drug delivery. Adv. Biochem. Eng. Biotechnol..

